# Is Regular Physical Activity Practice During a Submarine Patrol an Efficient Coping Strategy?

**DOI:** 10.3389/fpsyt.2021.704981

**Published:** 2021-07-12

**Authors:** Charles Martin-Krumm, Barbara Lefranc, Alan Moelo, Charlotte Poupon, Julien Pontis, Alexandre Vannier, Marion Trousselard

**Affiliations:** ^1^Ecole de Psychologues Praticiens (EPP), Paris, France; ^2^Institut de Recherche Biomédicale des Armées (IRBA), Bretigny sur Orge, France; ^3^APEMAC, Université de Lorraine, Nancy, France; ^4^French Military Health Service Academy, Paris, France; ^5^Ecole Camondo, Paris, France

**Keywords:** physical activity, military, thymia, exteroception, submarine patrol

## Abstract

**Introduction:** A nuclear-powered ballistic missile submarine (SSBN) is a singular professional environment, exposing personnel to isolation and confinement amidst sophisticated technology for the duration of a mission. Submariners see their mood and cognition deteriorate as their mission progresses. With regard to the benefits of physical activity (PA) on mental health, this study evaluates the impact of regular PA on the maintenance of thymia and sensory functioning during patrols.

**Method:** This pragmatic exploratory cohort follow-up study included 29 volunteer submariners before, during and 1 month after return from patrol. PA practice was evaluated by a daily self-questionnaire. This allowed submariners to be classified into two groups according to the median of the total duration in minutes of a sport practiced during the patrol (PA practicing submariners and non-practicing). Changes in mood and psychological activation, health (including sleep), unipodal stability, and accommodation distances were compared between the two groups over the period of the patrol.

**Results:** Overall thymic functioning deteriorated during the patrol. Submariners who practice PA maintain a stable level of activation unlike non-practicing PA submariners, but they exhibited both worse general health and sleep at recovery. For these personnel, postural control is better at the end of the patrol and far visual accommodation tends to be preserved.

**Conclusion:** PA during patrol alone is not sufficient to compensate for the thymic dysregulation induced by the SSBN environment. Nevertheless, it seems to help in maintaining an exteroceptive functioning. This exploratory study suggests directions for possible future research on physical activity associated with sensory stimulation amongst submariners, and more generally amongst people working in isolated and confined environments.

## Introduction

In the distant past, our ancestors lived with nature as cultivators, hunters, and gatherers. As technology and society evolved, people became increasingly sedentary. Their level of physical activity decreased. Then, for political, economic and/or military reasons—reasons that today might even include tourism which offers the wealthiest the opportunity to stay on an orbital base—the problem of physical inactivity has become increasingly acute. This is especially true of the exploration of extreme environments. Indeed, from the depths of the oceans to the confines of space, man can now confront extreme, unusual, isolated or confined conditions whose dangers, limited resources and constraints on the possibility of communicating and interacting with others, push professionals to the limit of their human functioning (1).

The American College of Sports Medicine [ACSM; (2)] recommends more than 5 days per week of moderate-intensity physical activity (PA), or either a combination of moderate to high-intensity PA or else 3 to 5 days per week of moderate to high-intensity PA. It is clear however that modern living environments accentuate sedentary lifestyles and their attendant deleterious effects, such as cardiovascular, psychological, or sleep-related difficulties. Moreover, PA has been shown to have beneficial effects, notably on the mental health of individuals (3, 4), or on cognitive functioning (5). These in turn affect the quality of relationships, health, and/or performance (6). Consequently, the quality of life of individuals depends partly on the practice of PA. Indeed, the World Health Organization (WHO, 2014) states that the absence of PA or a sedentary lifestyle, is the 4th risk factor for mortality worldwide and is among the main causes of breast and colon cancer, diabetes, and ischemic heart disease (7). Physical inactivity is therefore expected to be one of the main causes of mortality. The effects are already being felt in populations that are both increasingly overweight and whose physical performance on standardized tests is deteriorating (8). These phenomena have increased during the period of confinement imposed due to the pandemic (9): adults have gained weight (10), as have both children and adolescents (11).

Practicing a profession in an extreme, unusual, isolated, and/or confined environment exposes individuals to physical inactivity and weight gain and ultimately to a deterioration in both physical and mental health and body functioning. The nuclear-powered ballistic missile submarine (SSBN) is a very particular professional context that exposes submariners to an isolated and confined environment (ICE) and highly technological surroundings in which to live, during a patrol which may last 2 months or even more. This environment may adversely affect human psychology and physiology. Studying the impact of the SSBN environment on individuals highlights multiple constraints (confinement, isolation, promiscuity, watchkeeping rhythm, absence of natural light, an artificial atmosphere, exposure to constant noise) and suggests how these affect mood and physical states. The consequences can be particularly deleterious, so it is advisable not only to prepare individuals prior to missions and to provide for recovery after, but also to consider countermeasures, including the possibility of PA on board the vessel. Based on the benefits of PA to health, this survey assesses whether regular practice over the patrol helps to cope with psychological and body functioning. What do these countermeasures consist of? Do they actually limit the effects of being sedentary on psychological and body functioning? To what extent? What effects would they be?

PA is defined as any movement produced by skeletal muscles that is responsible for an increase in energy expenditure above resting expenditure (12). This definition applies to physical activities associated with work, leisure, sports, housework, and other components of daily life. The WHO defines four main areas of PA: activities in daily life, occupational activities, leisure and sports, and travel. PA is therefore plural, as it can be carried out in different contexts (professional, leisure, family, competition) and for different reasons (leisure, work, travel, performance). It is not to be confused with physical exercise and even less with sport. For the WHO, the distinction lies in the level of sophistication of the practice. PA includes physical activity in everyday life, at home, at work, traveling between places, and in non-competitive recreation. In the constricted space of a submarine with no possibility of escaping elsewhere, it will be most likely that PA occurs with the option of weight training or the use of a home-trainer.

PA can be practiced at different intensities: low, moderate, and high. Intensity is measured in METs (1 MET corresponds to the basic metabolic expenditure per Kg per minute of a subject, awake, at rest and sitting; 1 MET 3.5 ml O_2_.kg^−1^.min^−1^). According to the WHO, a moderate intensity PA (about 3-6 METs), requires a moderate effort and accelerates the heart rate (walking briskly, dancing, gardening, housework.). A high intensity PA (>6 METs) requires high effort, shortens the breath, and accelerates the heart rate considerably (running, swimming at a brisk pace, playing sports, and traditional games). It is important to note that the level of PA intensity, whether it is low, moderate, or high is really relative. That is, it is related to the abilities of people and the nature of their environment. The intensity depends on one's experience with PA, and one's physical condition. The crew members here are all in good health, practicing regular activity outside patrol periods. Of these, some continue to practice on board and some do not. In view of this, regular physical activity could be particularly beneficial on board an SSBN. This is a very special environment due to its recycled atmosphere and unusual, confined and isolated nature. During a patrol of several weeks underwater, as mentioned above, submariners operate in extreme and stressful surroundings. Their missions, or patrols, involve many unusual, often unnatural stimuli over a long period of time (13). Personnel are subjected to watches that disrupt the circadian rhythm (14). The environment is monotonous, and therefore provides a poverty of sensory modulations. A well-known effect of the patrols is the appearance of emotional and thymic disturbances. The work of Joly (15), Crosnier (16), and Lamour (17) on the adaptation of submariners to the constraints of an SSBN patrol describes the appearance of a syndrome of seasonal depression or “wintering” from mid-patrol. At the least there is an increase in negative and deterioration of positive moods, and a less restorative sleep (18). These effects are deleterious to cognitive functioning (16). Although there is a lack of objective data, submariners describe a disappearance of these thymic symptoms in the weeks following their return from mission. Recent data in the literature suggest that mood disorders involve a deterioration in interoceptive functioning (19, 20). It is interesting to note that while interoception has been well-studied in mood disorders, exteroception has received less attention. Exteroception is mediated by our five senses and allows us to perceive the outside world. Impaired exteroceptive functioning may be especially implicated in ICE because of the sensory characteristics of these particular environments. Initial results show a degradation of interoceptive (21) and exteroceptive performance (visual accommodation, olfactory detection, proprioception) during patrols (22). These findings prompt reflexions as to the relevant countermeasures to be deployed during missions.

The benefits of PA which have already been mentioned are also widely described in many psychiatric pathologies, particularly in the prevention and curative treatment of depression and anxiety (23). In healthy subjects, when practiced regularly, it is a recognized mental health factor (24). People who practice PA voluntarily are less anxious, less depressed, less neurotic (25). It improves stress tolerance and helps maintain cognitive abilities, such as memory and learning (26). Its benefits with regard to depression could lead to an improvement in exteroceptive functioning (20). In fact, the practice of PA implies *per se* exterior stimuli, especially those that are proprioceptive. PA appears to be a countermeasure of interest, simply applied and acceptable to personnel on ICE missions. It should be stressed that beneficial effects derive from voluntary activity. In the case of imposed exercise or efforts that are too intense, the effects are less, or even adverse (27).

However, to date, no study has evaluated the benefits of regular PA during SSBN patrols. We wished to explore the issue in order to deploy this simple countermeasure during ECI missions. The main objective of this study is to evaluate the impact of PA practice on thymic regulation during an SSBN patrol. Its secondary objective is to evaluate the impact of this practice on certain senses (visual accommodation and proprioception).

## Materials and Methods

### Design

Our study, a pragmatic exploratory cohort follow-up, was conducted on the crew of the SSBN *Le Triomphant* during an operational patrol in 2018. It was submitted to the South-East VI (Clermont-Ferrand) personal protection committee and received a favorable opinion on September 15, 2017 (study qualified as category 2: interventional research with minimal risks and constraints; IDRCB: 2017-A01329-44). An initial document was circulated to inform the personnel of the study to be conducted during the mission and its modalities. This document contained the regulatory aspects of the study as well as the contact details of both the promoter (Central Directorate of the Armed Forces Health Service) and the investigators. The ship's doctor then presented the study, its objectives and constraints during a briefing session with the crew. The different measures were planned over the duration of the mission (Table 1).

**Table 1:** Planning of measures.

	**Inclusion**	**Patrol**		**Recovery**
	**S1 or M1** ** (Baseline)**	**S2 or M2** ** (J10)**	**S3 or M3** ** (J50)**	**S4 or M4** ** (back + 1 month)**
Information	X			
Inclusion and exclusion criteria	X			
Consent	X			
Sports Tracking Logbook				
Type of sport and duration of practice	Daily			
Self-questionnaires				
Socio-demographic data	X			
Emotional state (SPANE)	X	X	X	X
Physiological thymic state (Thayer)	X	X	X	X
Enteroception (MAIA)	X	X	X	X
Health (GHQ, LEEDS, appetite)	X			X
Exteroceptive Tests				
Visual accommodation	X	X	X	X
Tummy tuck	X	X	X	X
Monopodial support	X	X	X	X
Stabilometry	X			X

*N = 29*.

### Participants

The population studied was that of the submariners of the SSBN *Le Triomphant*, red crew, scheduled for a 60-80-day patrol in the autumn/winter of 2017-2018 (the dates of SSBN missions are classified as defense secrets). The inclusion criteria are as follows: existence of an informed and written consent from the patient; affiliation to a social security system; subjects judged fit for underwater navigation according to the regulations in force within the Army Health Service (Ministerial Instruction n°500 on medical aptitude for underwater navigation). 29 volunteer submariners, all male, were included. Their average age was 29.8 years (SD: 6.45). Of these, 23 were living with a partner (76.67%) and 14 had at least one child (48.27%).

#### Professional Experience

The average length of service was 5,567 h on a nuclear attack submarine (NAS) (SD = 4,708) for the three submariners with NAS experience, and 6,835 h on an SSBN (SD = 6,505). The lowest work experience in the population was 0 h on NAS and 101 h on SSBN. The highest experience was 9,000 h on NAS and 25,000 h on SSBN. To illustrate what diving hours on an SSBN represent, an operational patrol corresponds to 2,000 h spent underwater. Three submariners from the population had never been on patrol on an SSBN before. For three other submariners, this was their last patrol.

#### Work Rate

In the population studied, eight submariners (27.6%) were on an “off-shift” schedule, working a day shift, with no night shift except for unforeseen interventions, and 20 (69%) were on a shift schedule (i.e., with a night shift every day) alternately between 8 P.M. and midnight, then between midnight and 4 A.M., and finally between 4 A.M. and 8 A.M. on the third night. One submariner did not specify his work rhythm.

#### Other Characteristics

Submariners reported an average weight before patrol of 75.96 kg [(60-97 kg); SD = 9.57]. Of 29 submariners, 24 (82.76%) were non-smokers. Two (6.89%) of the 29 submariners were left-handed.

### Measures

#### Physical Activity

A homemade questionnaire was given to the subjects to provide daily information on their physical activity during the patrol. They were asked to mention their daily PA, its nature, and duration. The participants completed the following questionnaire for each week of the mission to record their level of physical activity (Table 2).

**Table 2:** Frequency and type of Physical Activity week 1.

**Your physical activity week 1**			
	**Physical activity**	**Types of activities**	**Duration (min)**
D1 W1	Ergo cycle: ¨ Yes ¨ No		
	Other: ……………………………		
D2 W1	Ergo cycle: ¨ Yes ¨ No		
	Other: ……………………………		
D3 W1	Ergo cycle: ¨ Yes ¨ No		
	Other: ……………………………		
D4 W1	Ergo cycle: ¨ Yes ¨ No		
	Other: ……………………………		
D5 W1	Ergo cycle: ¨ Yes ¨ No		
	Other: ……………………………		
D6 W1	Ergo cycle: ¨ Yes ¨ No		
	Other: ……………………………		
D7 W1	Ergo cycle: ¨ Yes ¨ No		
	Other: ………………………………		

The descriptive statistics are presented in Table 3.

**Table 3:** Psychological variables description (Mean; Standard Deviation).

	**Baseline**		**D25**		**D55**		**Recovery**	
	**PA**	**N-PA**	**PA**	**N-PA**	**PA**	**N-PA**	**PA**	**N-PA**
**Thayer Questionnaire**								
GA	10.20 (2.073)	10.21 (2.08)	8.79 (2.86)	8.15 (2.91)	6.88 (3.76)	6.88 (3.76)	11.43 (2.03)	10.10 (1.73)
GD	4.40 (2.13)	3.86 (2.38)	4.46 (2.37)	5.07 (3.00)	5.25 (2.26)	5.50 (2.78)	4.43 (1.95)	3.80 (1.99)
IT	3.47 (3.18)	3.64 (3.05)	2.50 (3.13)	1.66 (2.01)	1.75 (2.38)	1.75 (1.78)	2.14 (2.63)	1.00 (1.56)
IR	8.20 (2.04)	7.43 (3.16)	9.21 (1.88)	8.08 (2.63)	8.92 (1.24)	7.13 (3.04)	8.86 (1.23)	8.70 (2.91)
**SPANE Questionnaire**								
PE	3.99 (0.67)	4.08 (0.44)	3.37 (0.68)	3.31 (0.68)	3.24 (0.97)	2.93 (0.92)	3.88 0(.86)	4.10 (0.35)
NE	2.38 (0.58)	2.40 (0.7)	2.06 (0.53)	2.00 (0.70)	1.89 (0.63)	2.11 (0.46)	1.92 (0.52)	1.82 (0.52)
**MAIA Questionnaire**								
TS	25.23 (4.53)	23.84 (3.32)	23.72 (5.57)	23.07 (3.78)	21.76 (5.17)	21.44 (3.88)	21.90 (6.54)	21.66 (4.64)
NCS	3.23 (0.78)	3.06 (0.75)	3.20 (0.9)	3.30 (0.85)	2.90 (1.11)	2.56 (0.83)	3.02 (1.12)	2.93 (1.05)
NDS	2.84 (0.69)	2.28 (0.49)	2.38 (0.79)	2.24 (0.63)	2.11 (0.85)	1.92 (0.59)	1.80 (0.78)	1.90 (0.83)
NWS	3.82 (0.60)	3.69 (0.92)	2.87 (0.77)	3.03 (0.67)	3.18 (0.53)	3.36 (0.83)	3.21 (0.91)	3.07 (0.77)
ARS	3.09 (0.86)	2.82 (0.78)	2.81 (1.12)	2.52 (0.65)	2.46 (1.05)	2.35 (0.93)	2.82 (1.03)	2.33 (0.95)
EAS	3.24 (0.85)	3.12 (0.63)	3.28 (0.94)	3.10 (0.95)	3.09 (0.92)	2.63 (0.79)	2.97 (1.24)	3.04 (1.04)
SRS	2.98 (0.91)	2.71 (1.01)	2.98 (0.99)	2.67 (0.95)	2.82 (1.03)	2.63 (1.27)	2.63 (1.43)	2.60 (1.28)
BLS	2.33 (1.11)	2.13 (1.04)	2.20 (1.13)	2.14 (1.01)	1.96 (0.81)	1.78 (1.18)	1.90 (1.08)	2.20 (1.34)
TrS	3.69 (1.22)	4.03 (0.92)	4.00 (1.28)	4.08 (0.92)	3.24 (1.52)	4.22 (0.90)	3.56 (1.64)	3.60 (1.07)

*PA, Physical Activity Group; N-PA, No-Physical Activity Group; SPANE, Scale of Positive and Negative Emotion; MAIA, Multidimensional of Interoception Awareness; GA, General Activation; DG, General Deactivation; IT, Inner Tense;IR, Inner Relaxation; TS, Total Score; NCs, Noticing Subscore; NDS, Not-Distracting Subscore; NWS, Not Worrying Subscore; ARS, Attention Regulation Subscore; EAS, Emotional Awareness Subscore; SRS, Self-Regulation Subscore; BLS, Body Listening Subscore; TrS, Trusting Subscore*.

#### Emotions

The 12-item scale of positive and negative experience questionnaire [SPANE, (28)] was used to assess subjective feelings of well-being, based on how frequently they were felt over the previous week.

#### Activation/Deactivation

The Thayer activation/deactivation questionnaire (29), comprising 20 items which the subject rates on a four-point Likert scale (1 = disagree to 4 = extremely agree) was used. Two sub-factors are calculated on an activation/deactivation axis (general activation: active, energetic, dynamic, fit, and general deactivation: sleepy, tired, attentive, drowsy) and two sub-factors on the tension/relaxation axis (inner tension: uneasy, worried, irritated, tense, and inner relaxation: indifferent, calm, relaxed, quiet). In the study, the questionnaire focused on the week that had just passed.

Several health indicators were also measured, general health, perceived sleep quality, exteroceptive variables.

#### General Health

The 12-item General Health Questionnaire [Goldberg GHQ-12, (30)] was employed. This is a questionnaire that explores the subject's psychosocial state over the past few weeks in terms of somatic complaints (or somatization), anxiety and insomnia, social dysfunction, and depression. Each item is associated with four response modalities which the subject rates on a scale of 0 to 1 (0 = not at all/no more than usual and 1 = a little more than usual/much more than usual). The overall score reflects the intensity of psychosocial distress experienced by an individual (Table 4).

**Table 4:** Health variables description (Mean; Standard deviation).

		**Physical activity**	**Baseline** ** M(SD)**		**Recovery M(SD)**	
			**PA**	**N-PA**	**PA**	**N-PA**
GHQ			1.93 (2.97)	1.57 (1.87)	5.36 (5.85)	1.50 (2.84)
Sleep	GTS	Difficult/easy	2.60 (2.32)	2.70 (2.23)	0.23 (3.37)	2.36 (3.59)
		Slow/Quick	2.07 (2.34)	2.57 (2.68)	−1.31 (3.52)	1.91 (3.81)
		Sleepiness	1.60 (2.53)	2.07 (2.50)	−0.31 (3.17)	0.82 (3.09)
	QoS	Restless	−2.00 (2.42)	−2.57 (2.31)	−2.85 (1.34)	−3.55 (1.51)
		wakeful periods	−0.40 (2.85)	0.43 (3.18)	−0.46 (3.13)	0.43 (3.18)
	AFS	Difficult/easy	1.00 (3.12)	1.07 (3.25)	−0.62 (3.38)	0.55 (3.14)
		Slow/Quick	1.27 (3.22)	1.71 (2.76)	−1.08 (3.28)	0.27 (2.53)
	BFW	Fatigue when wake up	1.93 (2.25)	1.71 (2.09)	−1.08 (3.35)	0.73 (2.97)
		Tiredness now	2.00 (1.96)	2.57 (1.83)	−0.62 (3.69)	2.36 (2.91)
		Balance and coordination	2.07 (2.25)	4.00 (0.78)	−0.08 (4.31)	1.91 (3.65)
Foodattitudes	Sensation of appetite		3.80 (0.94)	4.29 (0.73)	3.92 (0.49)	4.09 (0.54)
	Enjoy food		4.07 (0.59)	4.21 (0.43)	4.15 (0.55)	4.18 (0.60)
	Hunger when time to eat		3.80 (0.77)	4.14 (0.77)	3.62 (0.65)	3.91 (0.70)
	Satiety after eating		3.87 (0.52)	4.07 (0.47)	4 (0.10)	4.09 (0.30)
	Hunger between meals		2.80 (0.56)	3.36 (0.93)	3.77 (0.44)	3.64 (0.50)

*PA, Physical Activity Group; N-PA, No-Physical Activity Group; GHQ, General Health Questionnaire; GTS, Getting to sleep; QoS, Quality of Sleep; AFS, Awake Following Sleep; BFW, Behavior Following Wakening*.

#### Perceived Quality of Sleep

The Leeds Sleep Evaluation Questionnaire (31) (LEEDS) with visual analog scales assessed four aspects of sleep evaluation: sleep quality and degree of sleepiness, sleep quality, wake quality, and post-wake quality, and performance. It makes it possible to monitor subjective changes in sleep with respect to usual subjective sleep. A low score indicates that sleep is worse than usual.

#### Exteroceptive Variables

Proprioception was assessed using postural stability tested with a stabilometric static platform (Stabilotest). Subjects were asked to stand for 1 min with their eyes open, and 1 min with their eyes closed. Among the numerous metrics regularly used to characterize postural stability (32, 33) in the literature, we assessed the most frequent metrics: weight distribution between left and right foot, sway length, standard deviation along the sagittal (Antero-posterior) and frontal (Medio-Lateral) directions, 90 confidence elliptic area, and slope its principal axis, Center of pression (CoP) mean velocity, and its variance. Vision was assessed by the Parinaud scale (the French equivalent to the Jaeger chart), which measures the natural accommodation distance (from the tip of the nose to the reading surface, measured with a tape measure in cm) at which the subject holds a text to read it comfortably. Paragraphs of text are written in decreasing font size. The recommended reading distance to test visual acuity is 33 cm, with a tolerance ranging from 30 to 35cm. Luminance was controlled with a luxmeter to ensure that the lighting environment was the same for all subjects. Moreover, accommodation evaluation was assessed for natural accommodation, and both maximal and minimal accommodation. The measurement was made from the tip of the nose to the reading surface, using a tape measure. Individuals kept their glasses on if they usually wore them. Proprioception (32, 33), mediated by balance, was measured with the unipodal standing test (UPST), over three successive series by recording the maximum time of maintaining posture on the preferred foot, eyes open (EO) then eyes closed (EC). The average of the times and the best time achieved for each of the two situations were retained.

#### Experimental Procedure

**Figure 1:**
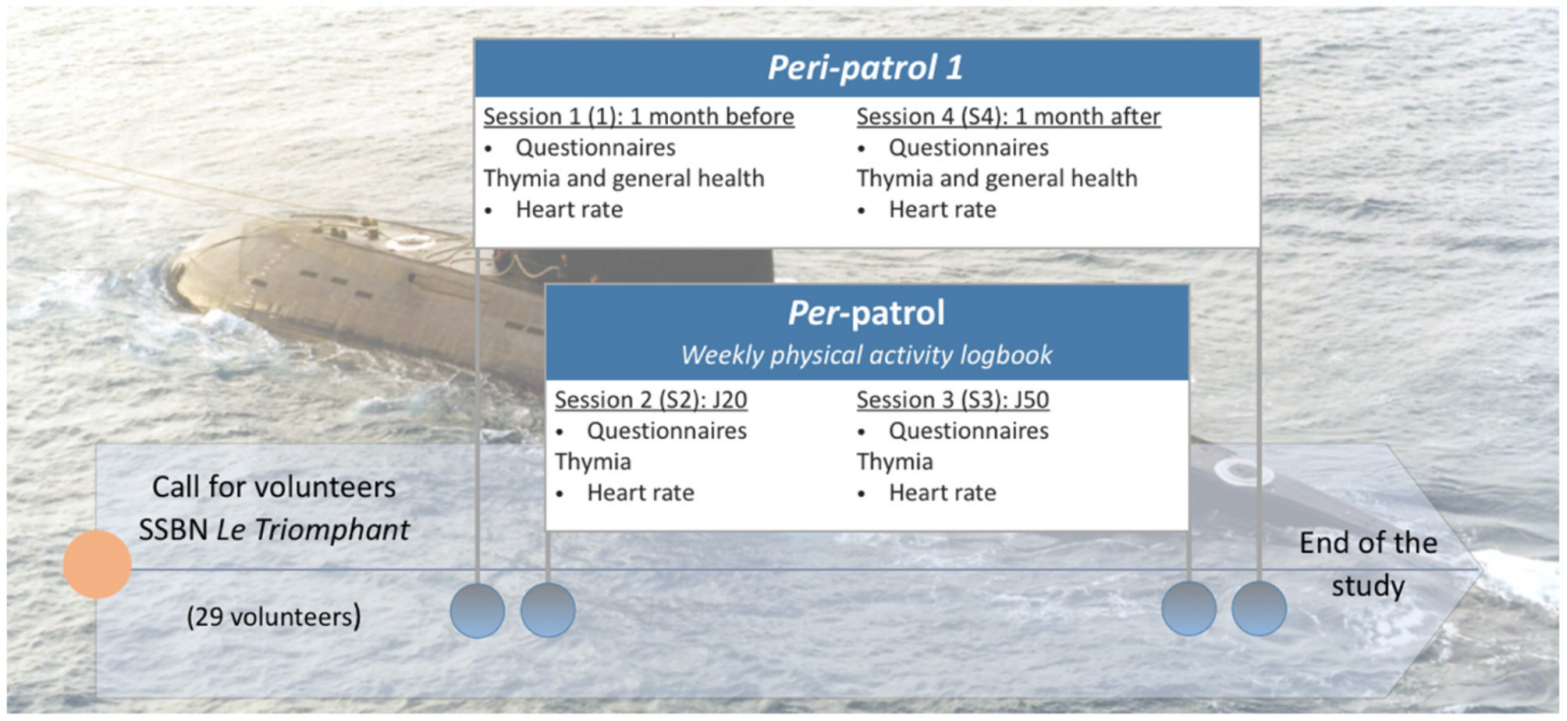
Proposed experimental design according to operational constraints. The indiced collection times, day 10 and day 50, are approximations.

The experimental design of the study is described in Figure 1.

Data were collected in four stages: before, during (D10 and D50 of the patrol) and 1 month after the return from the patrol, with the exception of the health variables collected before and on return from the patrol.

### Data Analysis

Statistical analysis of the data set was carried out using IBM SPSS Statistics 25 software for some analyses). Data will be expressed in number and proportion (%) for the qualitative variables or modalities (stress events: yes/no) and in mean (M) and standard deviation (SD) for the quantitative variables. A categorization of the subjects according to the importance of physical activity was made on the basis of the median of the total duration in minutes of declared sports practice during the patrol whatever the nature of the PA. This categorization describes two groups: a group of so-called PA practicing submariners with a total duration of practice during the patrol greater than or equal to the median of the included population, and a group of so-called non-practicing submariners, with a total duration less than the median of the included population.

For the pre-departure collection, the percentages between the two groups were compared using the Chi-2 Test and Fisher's Exact Test when the Chi-2 conditions were not met. Group means were compared using the Student's *t*-test for independent samples (PA and non-PA status) when the variables in the samples compared followed a normal distribution. For the evaluation of the impact of the patrol on the two groups (sport and non-sport status), analyses of variance (ANOVA) on the variables collected during the four measurement sessions [S1 before, during (S2 and S3, D10 and D50 of the patrol, respectively) and S4 1 month after the return from the patrol] were performed. Tukey *post-hoc* tests were used when necessary. For the variables collected at the S1 inclusion and S4 recovery phases, the means of the before-and-after deltas were compared using the Student's *t*-test for independent samples when the variables of the samples compared followed a normal distribution. A difference was considered significant at *p* < 0.05. A trend toward a significant difference was noted when 0.05 < *p* ≤ 0.10.

## Results

### Description of Physical Activities During the Patrol

Three types of sports, determined by the limited space available in the submarine, can be practiced during the mission: ergocycle, treadmill, and weight training (Table 5). Of the entire patrol, 6 submariners did <1 PA session per week, 10 did between 1 and 3 sessions, and 9 did more than 3 sessions. Two submariners stated that they did not practice any PA. One submariner did not fill in his PA record book. Over the first 60 days of diving analyzed, the average number of days of PA performed was 21.1 (SD = 17.5). Over the first 30 days, the average was 9.8 and over the next 30 days, 10.5. The average time spent on PA during the patrol was 1220.2 min (SD = 1560.8) with a median value of 620 min. Of the 28 subjects who filled in the PA logbook, 15 reported more than 620 min of practice during the patrol, one subject reported 620 min and 12 reported <620 min. Sixteen subjects were therefore considered to belong to the group of so-called PA practicing submariners. They reported an average of 34.33 days with PA (SD = 14.11). Their minimum number of days with PA was 16, and their maximum was 55. Non-practicing submariners reported an average of 5.93 days with PA (SD = 5.34). Their minimum number of days with PA was 0, and their maximum was 17.

**Table 5:** Types of activities chosen and number of participants.

**Type of activities**	**Number of participants**
Sailors who prefer cycling	7
Sailors preferring to run on the treadmill	2
Other activities	17
Total	26

### Impact of Sport/Non-sport Status at Inclusion

The ship's doctor was in charge of all the measurements during the patrol in which the study was carried out, which limited the potential bias related to measurement errors that could have occurred if there had been several experimenters. Thus, the study protocol was scrupulously followed.

### Socio-Demographics

There was no difference in age between the two groups (t = −1.51, *p* = 0.14). There was no difference between the PA/non-PA group in terms of family situation (single or couple; *p* = 0.21), reported stress event (*p* = 0.55), and smoking outside of patrol (*p* = 0.56). No difference was found in terms of professional experience (*p* = 0.85 and, *p* = 0.18, respectively, for patrol hours in NAS and SSBN). There was no difference between the two groups in terms of work rhythm on board, watch or off watch (*p* = 0.28).

### Psychopathological Functioning

Concerning the thymic state, no difference was found between the two groups on the psychological variables collected in the Thayer and SPANE questionnaires. Concerning health, no difference in terms of weight, appetite or GHQ score was found between the two groups (respectively, *p* = 0.48 and *p* = 0.69). There was no significant difference in sleep between the two groups at inclusion, except for the higher sleep disturbance rating with respect to usual subjective sleep in PA practicing submariners (M = 2.07, SD = 2.25) compared to non-practicing (M = 4, SD = 0.78, *p* = 0.01).

### Exteroceptive Functioning

Regarding visual accommodation, there was a difference in distribution between the two groups for the distance of accommodation before the patrol (U = 6; *p* = 0.017). Subjects in the non-PA group were distributed toward low values of the distribution interval of the “distance of distant accommodation,” whereas subjects in the PA group were distributed toward high values. Regarding proprioception, no difference was found on the unipodal station test.

### Impact of Patrolling on Psychological Functioning According to PA/Non-PA Status

The SPANE scores on the two subscales designed to assess positive and negative emotions were used.

There was an effect of the data collection session on the positive emotion subscale score (*p* < 0.001), with no effect of sport status (*p* = 0.68), nor interaction (*p* = 0.14) between the session and group factors. *Post-hoc* analyses showed that the positive emotion score was highest at the inclusion session (S1) and the recovery session (S4) compared to the per-patrol sessions at D10 (S2) and D50 (S3). The positive emotion score was higher at D10 compared to D50.

A session effect was also found on the negative emotion subscale score (*p* = 0.01), with no effect of PA status or interaction between session and group factors (*p* = 0.92 and *p* = 0.86, respectively). *Post-hoc* analyses showed that the negative emotion score was significantly higher at the inclusion session (S1) compared to the per-patrol sessions of D10 (S2) and D50 (S3) and recovery (S4). Negative emotion scores showed no difference between the per-patrol sessions of D10 (S2) and D50 (S3) and recovery (S4).

The Thayer questionnaire subscales were used to assess general activation and general deactivation in the analysis below.

A session effect and a PA status effect on the general activation subscore were found (*p* = 0.005 and *p* = 0.001, respectively), without interaction between the session-PA status factors (*F* = 1.63, *p* = 0.20). The PA practicing submariners exhibited a higher activation level at each of the sessions (Table 6).

**Table 6:** Analyses for the interaction between the variables and the condition, PA vs. Non-PA.

**Variables x status**	**F ratio**	**p**	**Partial η^2^**	**Alpha = 0.05**
**Thayer**				
Activation	1.63	0.20	0.07	0.41
General deactivation	4.45	0.001	0.18	0.87
Internal tension	1.18	0.32	0.05	0.31
Relaxation	1.38	0.32	0.01	0.11
Monopodal stance	5.38	0.001	0.28	0.92

*N = 29*.

A session effect on the general deactivation subscore was found (*F* = 8.51, *p* < 0.01), and an interaction between the session-sport status factors (*F* = 4.45, *p* < 0.001). The general deactivation subscore deteriorated significantly during the patrol, with no effect of PA status (*p* = 0.40). *Post-hoc* analyses showed that the general deactivation score was highest at D50 for non-PA practicing compared to PA practicing (*p* = 0.05). A “session” effect on the internal tension subscore (Thayer questionnaire) was also found (*F* = 4.54, *p* < 0.01), with no effect of PA status (*p* = 0.85), nor interaction (*p* = 0.32). *Post-hoc* analyses showed that internal tension was highest during the inclusion session before the patrol compared to the levels at D10, D50 and after recovery (Table 1).

There was no session effect, no group effect, and no interaction for the relaxation sub-score.

### Health

The results show a significant difference between the two groups on the delta recovery-inclusion GHQ scores (t = −2.45, *p* = 0.02). The PA practicing submariners had a higher GHQ score 1 month after the mission compared to inclusion (M = +3.18, SD = 4.07) whereas it decreased in the non-PA ones (M = −0.2, SD = 1.62). We also found a trend toward a difference between the two groups on the recovery-inclusion (delta) for the sleep length subscores of the Leeds questionnaire. The latter decreased in PA practicing men after recovery compared to inclusion in contrast to non-PA practicing (*p* = 0.09).

### Exteroception

Regarding far accommodation, the analyses showed a trend toward a difference in position between the two groups 1 month after the end of the patrol (recovery) (U = 6; *p* = 0.017). As with inclusion, the non-PA group were distributed toward low values of the far accommodation distance, whereas subjects in the PA group were distributed over the whole range of accommodation distance distribution.

Regarding proprioception, there was an effect of session (*F* = 6.24, *p* < 0.01) on monopodal stance duration, but no effect of sport status (*F* = 0.13, *p* = 0.73). There was an interaction (*F* = 5.38, *p* < 0.01) between the session and group factors. *Post-hoc* analyses showed that the duration of monopodal support was higher in the non-sportsmen at the inclusion session, compared to the following sessions. There was no significant difference between sessions in the sportsmen.

## Discussion

As in previous studies evaluating the mood and health of submariners during an SSBN patrol, we found a deterioration in thymic functioning during the patrol. This was associated with a more or less complete recovery 1 month after return, depending on the variables and the sport group.

The results obtained do not allow us to confirm the main hypothesis of a positive impact of PA practice on mood. Although no difference was observed between the PA and non-PA practicing groups in terms of positive and negative emotions, the deterioration in the activation state during the patrol for the non-PA submariners suggests that the patrol is not subjectively experienced in the same way by the two groups. This result suggests that regular practice of PA while on patrol would allow for better vigilance, which is recognized as an important factor for boat safety.

These results should be considered in the light of the differences in subjective sleep quality and general health observed 1 month after the patrol between the two PA groups: compared to inclusion, the PA practicing submariners slept less and expressed a poorer general health functioning. Although these data alone are in agreement with existing studies (34), the results as a whole do not provide a clear answer to the hypothesis of better health maintenance in the broad sense for submariners who regularly engage in PA. It would be interesting to compare the two groups in terms of their personality profile and their strategies for dealing with stress. It has been observed that army soldiers, even with addictive behavior, practice PA more intensively in stressful situations. During a six-month projection in Afghanistan (raptor 201 mandate), PA was an effective means of adapting to operational stressors and managing situational anxiety (34). These data suggest that PA practicing submariners might use it cognitively as a way to better manage the stresses of patrol. PA could then be considered for sport submariners as an obligatory practice rather than an activity for pleasure. As far as humans are concerned, a compulsory PA practice is known to trigger a stress reaction (35, 36). During the patrol, physical practice for sport submariners could be a means to deal with anxiety and stress. Taking this status into account may be necessary when determining the training programs for regular sportsmen. It is important to emphasize that mindfulness-based interventions may reduce anxiety and render a given activity more pleasurable than under normal circumstances through the reallocation of attention toward bodily signals (37). Such an approach could also develop a desire for PA during patrol among non-athletic submariners.

Furthermore, unipodal stability data and its evolution suggest that PA and non-PA practicing submariners do not control their posture in the same way. Maintaining the standing position is a biomechanical and neurophysiological feat for humans. The human body has developed a system of postural regulation, not only so as to stand upright despite external constraints (sloping or slippery ground, etc.) or internal constraints (breathing, blood circulation, etc.), but also to orient itself in space and prepare its movements with precision. This is particularly relevant in the environment of an SSBN on a mission. Our study shows that between PA and non-PA practicing subjects, it is only in non-PA submariners that unipodal stability deteriorates while on patrol. In addition, we found a distribution of remote accommodation distances around smaller distances. The interaction between these exteroceptive differences needs to be further explored: in submarines, where the viewing distance is limited, distant accommodation is very rarely used.

Further studies concerning the other exteroceptive sensors which are differently stimulated in a mission environment, would help to confirm our initial results.

This exploratory study has several limitations. Firstly, the low number of volunteers reflects a possible recruitment bias. Where results are borderline, they probably have minimal significance. Given the multiplicity of tests carried out, discussion may be needed to determine which of the observed results should carry weight. Secondly, the choice of psychological scales has been validated for the exploration of mood disorders, but there are other tests that focus more specifically on seasonal depression. These questionnaires are subjective and prone to self-measurement bias. Further complementary psychological assessments, including personality tests to determine an anxious-trait and coping profile, would have helped to describe better the impact of regular sport during a patrol. Finally, our study classifies practitioners according to a duration of PA while on patrol. However, we have no evidence that submariners practicing PA on board, practice it on land and vice versa. The classification into PA and non-PA practicing is contextual and does not necessarily reflect the PA level of an individual.

This study is one of the first to assess the role of PA in a confined and isolated environment, such as an SSBN. This is a very specific environment that seems ideal for exploring all the issues of individual adaptation in extreme and stressful surroundings. The interior of a submarine is a rigorous environment in which human error can jeopardize a mission and an entire crew. For this reason, submariners are carefully selected. They are all in excellent physical and mental health, which reduces confounding biases on the health status of individuals, but also makes it difficult to extrapolate the results to any other population. Nevertheless, it is conceivable that if differences are observed in this sample where differences between individuals are minimal, they would be greater in the general population. Finally, it should be noted that dividing the groups after the fact makes it possible to limit measurement bias. The daily filling-in of a diary limits the bias that having to recall details later would introduce.

## Conclusion

Although regular PA is associated with better activation adaptation on an SSBN during a patrol, this countermeasure does not seem sufficient to limit the thymic and health impact on professionals of the constraints imposed by such an isolated and confined environment. PA could nevertheless play a role in maintaining exteroceptive sensory skills while on patrol. Enhancing it with artificially increased sensory stimulation might be more beneficial than mere practice alone. An implementation of pleasure practice could be useful for thymic regulation. It would be interesting to conduct a study on this subject so as to understand better the dissociation between the improvement in both physical functioning and activation, and the absence of benefits for thymia and health. It is possible to imagine that, in the long term, a crew member in charge of a specific role on board could be trained to propose physical activity programs dedicated to this population and this type of environment. Such programs remain to be imagined and their effects to be evaluated. In order to improve the efficiency of ships and to maintain the good health of those on board, it would seem necessary to evaluate other countermeasures to the psychic effects observed during SSBN patrols. The results of any such evaluations would be of great use to professionals working in extreme, unusual, isolated and/or confined environments such as space or polar stations.

## Data Availability Statement

The datasets presented in this article are not readily available because Confidential data linked to the Frenchs Forces. Requests to access the datasets should be directed to marion.trousselard@gmail.com.

## Ethics Statement

The studies involving human participants were reviewed and approved by Comité de Protection des Personnes sud-est VI (France) (IDRCB: 2017-A01329-44). The patients/participants provided their written informed consent to participate in this study.

## Author Contributions

CM-K wrote the article, contributed to the data collection, and the methodological aspects. BL, AM, CP, JP, AV, and MT have made substantial contribution to the conception of the design of the protocol, gave feedback to the manuscript. BL particularly contributed to the data analysis and the redaction of the manuscript. AM, CP, and JP particularly contributed to the data collection. MT particularly contributed to the conception of the study's design, the data analysis, and the redaction of the manuscript. BL, AM, CP, JP, AV, and MT have read and approved the submitted version of the manuscript.

## Conflict of Interest

The authors declare that the research was conducted in the absence of any commercial or financial relationships that could be construed as a potential conflict of interest.
